# The antibacterial effect of non-thermal atmospheric pressure plasma treatment of titanium surfaces according to the bacterial wall structure

**DOI:** 10.1038/s41598-019-39414-9

**Published:** 2019-02-13

**Authors:** Myung-Jin Lee, Jae-Sung Kwon, Heng Bo Jiang, Eun Ha Choi, Gyungsoon Park, Kwang-Mahn Kim

**Affiliations:** 1 BK21 PLUS Project, Department and Research Institute of Dental Biomaterials and Bioengineering, Yonsei University College of Dentistry, Seoul, 03722 Korea; 2 School of Stomatology, Taishan Medical University, Tai’an, Shandong 271000 China; 3 Plasma Bioscience Research Center, Kwangwoon University, Seoul, 01897 Korea

## Abstract

Titanium is commonly used as a biomaterial for dental implants. In this study, we investigated the antibacterial properties of titanium samples following treatment with a non-thermal atmospheric pressure plasma jet (NTAPPJ) on bacteria with two different cell wall structures, including gram-positive and gram-negative bacteria. The hydrophilicity and surface energy of titanium surfaces were significantly increased after NTAPPJ treatment without altering topographical features. Changes in the chemical composition and reductive potential were observed on the NTAPPJ-treated titanium surfaces. The adhesion and biofilm formation rate of bacteria were significantly reduced on the NTAPPJ-treated titanium surfaces compared with the untreated samples, which was confirmed by fluorescent imaging. Regarding the comparison between gram-positive and gram-negative bacteria, both adhesion and the biofilm formation rate were significantly lower for gram-negative bacteria than gram-positive bacteria on samples treated for longer durations with the NTAPPJ. Transmission electron microscopy imaging showed a comparably more disruptive membrane structure of gram-negative bacteria than gram-positive bacteria on the NTAPPJ-treated surfaces. Our results indicated that the NTAPPJ treatment could be useful for preventing bacterial adhesion and biofilm formation on titanium dental implant surfaces, while the reductive potential on surfaces treated by the NTAPPJ could cause oxidation of bacteria, which could be more sensitive to gram-negative bacteria due to differences in the cell wall structure.

## Introduction

Dental implants are a key tool used to facilitate the prosthetic replacement of missing teeth in the field of dentistry^1^. Dental implants are commonly made from titanium, and although dental implants have achieved notable success, limitations, such as infections, remain a challenge^2,3^. These types of infections cause serious complications in patients and pose great challenge to clinicians^4,5^. Bacterial adhesion and subsequent growth of bacteria on the implant surface cause the formation of a biofilm, which is one of the main reasons for implant failure^6,7^. In addition, the ability of bacteria to form biofilms is the most relevant factor in the pathogenesis of inflammation surrounding the implant and inflammation of periodontal tissues, commonly known as peri-implantitis and periodontitis, repsectively^8^. Therefore, it is crucial to prevent bacterial biofilm formation, and many investigations have focused on the development of biomaterials with infection-resistant surfaces^9^.

Several attempts have been made to inhibit bacterial adhesion and consequently prevent the possibility of implant failure by using chemical or physical methodologies^10^. The inactivation of bacteria can be achieved by chemical and/or physical means, such as heat (steam or dry autoclaves), chemical treatment (active gases, such as chlorine or ozone), and irradiation (ultraviolet or gamma)^11^. However, most of these conventional techniques can cause damage to the treated substrate^11,12^. Therefore, alternative methods are needed, and non-thermal atmospheric pressure plasma could be a potential tool for substituting conventional methods for the control of bacterial biofilm formation.

Recently, numerous studies regarding the role of non-thermal atmospheric pressure plasma jets on the antibacterial properties of biomaterial surfaces have been reported^13,14^. Numerous studies have directly killed bacteria using NTAPPJs^13,15–17^. However, few studies have considered the inhibition of bacterial adhesion by surface modification using NTAPPJs. Previous studies have indicated that the treatment of titanium surfaces with an NTAPPJ causes antibacterial effects^18,19^. Because the initial interaction between bacteria and a biomaterial takes place on the surface, plasma treatment of a biomaterial may cause different outcomes in inhibition efficiency between two distinctive types of bacteria^13^. Nonetheless, only a tentative theoretical explanation has been proposed. Another study has investigated the mechanism of surface changes and the effect on bacterial inhibition, particularly for bacteria with different cell wall structures.

Therefore, this study aimed to investigate the effect of NTAPPJ treatment on the surface properties of titanium, which is a commonly used material for dental implants, and the consequential influence on the adhesion of bacteria with two different cell wall structures (gram-positive and gram-negative bacteria). The null hypothesis was as follows: 1) there is no difference in the chemical change of the titanium surface after NTAPPJ treatment, and 2) there is no difference in the antibacterial effects against gram-positive and gram-negative bacteria when they are cultured on NTAPPJ-treated titanium surfaces.

## Methods

### Preparation of titanium samples

Commercial pure titanium disks with a 10 mm diameter and 2 mm thickness were used in this study. The disks used in all experiments described below were mirror-polished with #400, #600, #800, #1500, and #2000 grit paper sequentially and ultrasonically cleaned with acetone, ethyl alcohol, and distilled water for 15 min each. All samples were sterilized in an autoclave (121 °C for 15 min).

### Treatment with the NTAPPJ

The non-thermal atmospheric pressure plasma jet (NTAPPJ) device was provided by Kwangwoon University (Plasma Bioscience Research Center, Kwangwoon University, Korea), and its structure has been well described in previous studies^20,21^. Plasma was generated using compressed air gas (5 L/min flow rate) and by applying a 15 kV voltage and a 13 mA current. Each of the test samples was treated with the NTAPPJ for 2 and 10 min, and the distance between the tip of the plasma flame and the sample was 3 mm. Samples that were not exposed to the plasma were used as controls. Each test and control group was designated as NP (control group), P2 and P10.

### Surface characterization

The surface roughness of the test and control groups was examined by optical surface profilometry (ContourGT, Bruker, Tucson, AZ, USA). Roughness parameters (Ra and Sa) were obtained as 2D and 3D roughness parameters, respectively. The optical profilometer measured the surface height and represented the surface height in grayscale format. The sample was measured at a magnification of 10× with a scanning area of 231 × 173 µm.

Changes in the hydrophilicity and energy of the titanium surface were measured using a video contact angle goniometer (Phoenix 300, SEO, Gyeonggi-do, Korea) with Image XP software (SEO) by dropping 8 µL distilled water and ethylene glycol (Sigma-Aldrich) on NTAPPJ-treated and untreated titanium surfaces. After 5 s, the static contact angle was measured, and the average of the values on the right and left sides was reported^22,23^. The advancing and receding contact angles were evaluated at an inclination of 90° according to the Owens-Wendt method^24^. The test was conducted at a room temperature of 20 °C.

The chemical composition of the titanium surfaces was evaluated using X-ray photoelectron spectroscopy (XPS; K-alpha, Thermo VG Scientific, Waltham, MA, USA) with a monochromatic A1 Kα source (1486.6 eV). All samples were prepared as previously described (mirror-polished with #400, #600, #800, #1500, and #2000 grit SiC paper sequentially and ultrasonically cleaned with acetone, ethyl alcohol, and distilled water for 15 min each). The surfaces of the control samples were cleaned by low energy Ar plasma in a vacuum environment before XPS analysis. For the experimental groups, samples were treated with the NTAPPJ and then cleaned by low energy Ar plasma in a vacuum environment before XPS analysis. XPS analysis was then performed to analyze the chemical bond energy changes caused by the NTAPPJ treatment. The anatomic compositions of C, O, N, and Ti were analyzed to evaluate the change in surface chemistry before and after exposure to the NTAPPJ. The resolution of the spectra was 0.78 eV, based on the full width at half of the maximum of the Ag 3d5/2 peak in a standard Ag specimen. The C1s peak at 284.8 eV was used as a reference.

Additionally, to characterize the plasma-treated Ti surface, we used water-soluble tetrazolium (WST). WST was prepared using WST solution (EZ-Cytox, Daeil Lab Service, Seoul, Korea) containing 0.9% NaCl solution at a volumetric ratio of 1:10. The optical density (OD) of WST was maintained at approximately 1.5 using a pure magnesium block. One hundred microliters of WST that was calibrated to an OD of 1.5 was dropped onto the Ti surface, followed by drip-sucking 5 times. The OD value of the WST solutions was measured at a wavelength 450 nm with a microplate spectrophotometer (BioTek, Winooski, VT, USA).

### Bacterial strains and culture conditions

Clinical strains were isolated from Korean individuals and obtained from the Korean Collection for Oral Microbiology (KCOM, Gwangju, Korea). Four bacterial strains were used, which included two gram-positive bacteria, namely, *Streptococcus mutans* (KCOM 1054) and *Staphylococcus aureus* (KCOM 1025), and two gram-negative bacteria, namely, *Klebsiella oxytoca* (KCOM 1569) and *Klebsiella pneumoniae* (KCOM 2770). All strains were cultured and maintained in brain heart infusion (BHI) broth or agar plates in an incubator at 37 °C. BHI broth was used to dilute the culture until the concentration reached approximately 1 × 10^8^ CFU/mL.

### Evaluation of bacterial colony forming units (CFUs)

For the adhesion experiment, 1 mL of bacterial culture suspension for each bacterial strain was placed on the titanium specimens treated or untreated with the NTAPPJ and incubated at 37 °C for 24 h under aerobic conditions. After incubation, the titanium specimens were gently washed twice with phosphate-buffered saline (PBS) to remove any nonadherent bacteria, and attached bacteria were then harvested in 1 ml BHI by sonication (Ultrasonic Cleaner SH-2100; Saehan Ultrasonic) for 5 min. One hundred microliters of the harvested bacterial suspension was spread onto a solid agar plate and incubated for 24 h at 5% CO_2_ and 37 °C. The total number of colonies was then counted.

### Biofilm formation assay

The ability of bacteria to develop biofilms was assessed by staining attached cells with crystal violet as described previously^25–27^. A bacterial suspension (1 × 10^8^ CFU/mL, 500 μL) was seeded onto titanium specimens and incubated at 37 °C for 24 h to allow biofilm formation. The nonadherent bacteria were removed with a pipette, and the specimen was washed three times using 5 mL PBS. A 0.1% crystal violet solution was used to determine biofilm formation on the surface of the titanium discs. The PBS-washed titanium discs were placed into a 12-well plate, submerged in 1 mL of crystal violet solution, and incubated at room temperature for 10 min. The discs were then transferred into a new 12-well plate and rinsed three times with 5 mL PBS to remove excess crystal violet solution. To elute the crystal violet, 500 μL of 30% acetic acid was added, and the plate was incubated at room temperature on an orbital shaker (Biofree, Buchen-si, Gyeonggi-do, Korea) at 250 rpm for 15 min. One hundred microliters of acetic acid solution containing the crystal violet stain retained by the biofilms was added to each well of a 96-well plate (SPL, Pochein-Si, Gyeonggi-Do, Korea), and the amount of biofilm that developed was measured at a wavelength of 595 nm using an ELISA reader (Epoch, Biotek, Winooski, VT, USA).

### Bacterial viability assay

To analyze the viability of adherent bacteria, each bacterial strain that was placed on the NTAPPJ-treated or untreated titanium as described above was stained using a live/dead bacterial viability kit (SYTO9 and propidium iodide, Molecular Probes, USA) according to the manufacturer’s protocols. Equal volumes of SYTO9 dye and propidium iodide were mixed thoroughly. Three microliters of the mixture was added per 1 ml of bacterial suspension. After 15 min of incubation at room temperature in the dark, the stained samples were observed using a confocal laser microscope (LSM700, Carl Zeiss, Thornwood, NY, USA).

### Analysis of bacterial morphology

The morphological changes in four bacterial strains cultured on plasma-treated and untreated titanium surfaces were examined using scanning electron microscopy (SEM; FE SEM S-800, Hitachi, Tokyo, Japan) and transmission electron microscopy (JEM-1011, JEOL, Japan). To prepare samples for SEM, 1 mL of the bacterial suspension (10^8^ cells/mL) was added to the titanium specimen in a 24-well plate and incubated for 24 h. The samples were then washed twice with PBS before fixing with 2% glutaraldehyde-paraformaldehyde in 0.1 M phosphate buffer (pH 7.4) for at least 30 min at room temperature. The samples were then postfixed with 1% OsO_4_ dissolved in 0.1 M PBS for 2 h, dehydrated in a gradually ascending series of ethanol, treated with isoamyl acetate, and subjected to a critical point dryer (Leica EM CPD300, Wien, Austria). The samples were coated with Pt (5 nm) using an ion coater (Leica EM ACE600, Wien, Austria) and examined and photographed using a scanning electron microscope at an accelerating voltage of 2 keV.

### Statistical analysis

All statistical analyses were performed using IBM SPSS software version 21.0 (IBM Korea Inc., Seoul, Korea) for Windows. All experiments were performed in triplicate for each timepoint and repeated three times. The results between three groups (NP, P2, and P10) at each timepoint were analyzed by one-way analysis of variance (ANOVA) with Tukey’s test. Differences with *P*-values less than 0.05 were considered statistically significant.

## Results

### Surface characterization after NTPPJ treatment

**Figure 1:**
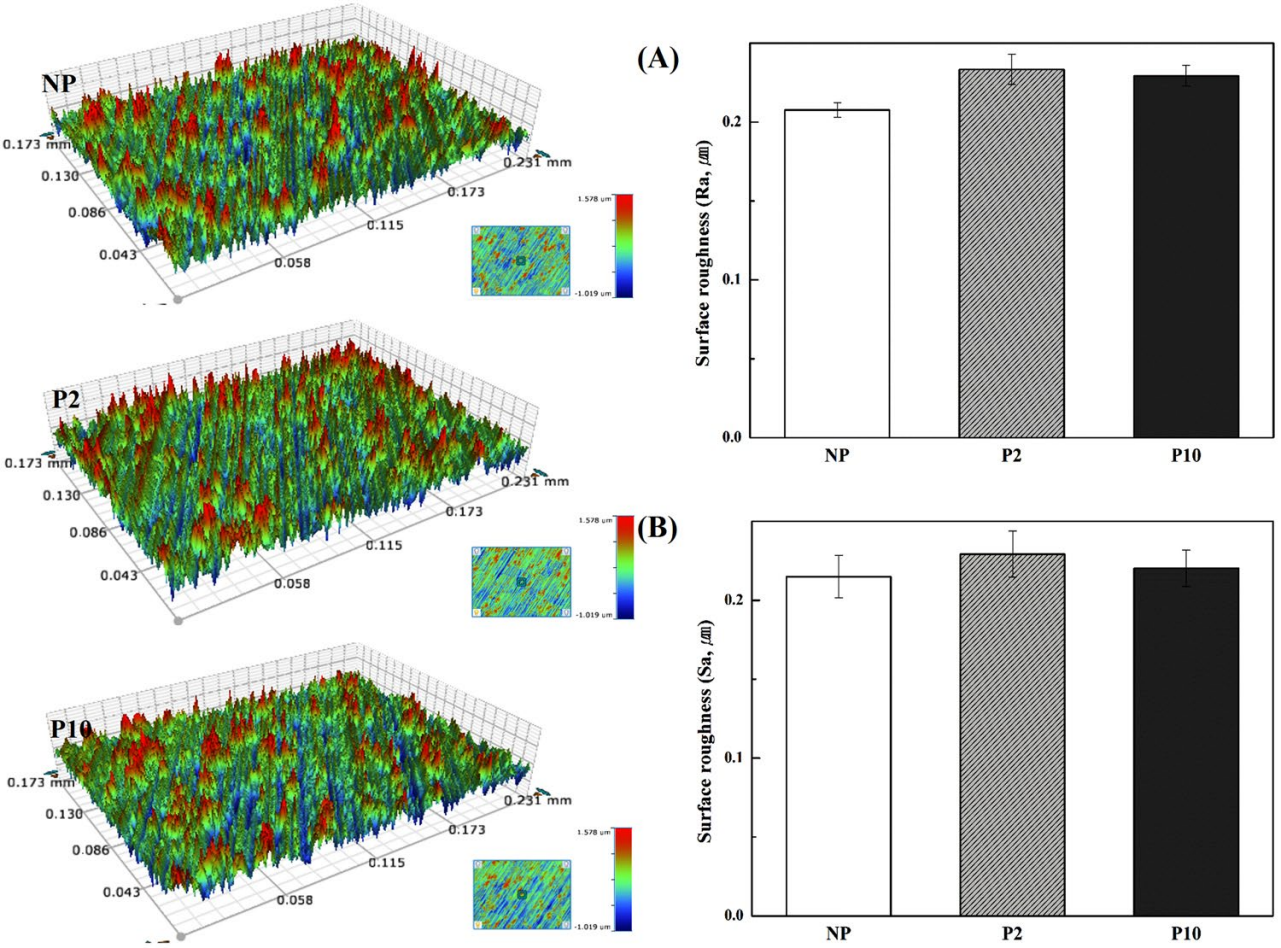
Representative three-dimensional (3D) surface topographic images of the titanium disc surface; NP, P2, and P10. Surface roughness parameters, (**A**) Ra and (**B**) Sa, were quantitatively measured.

**Figure 2:**
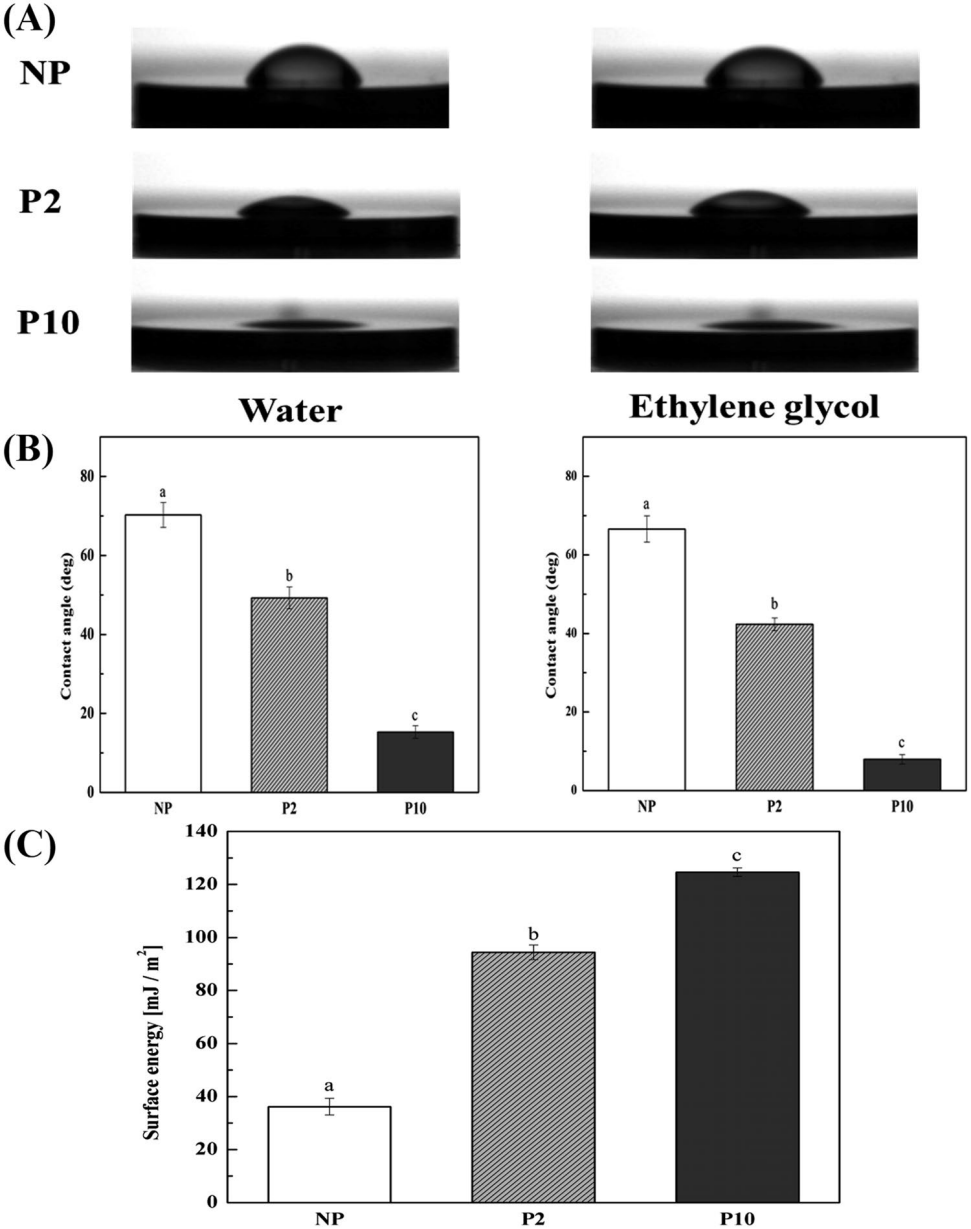
Changes in the hydrophilicity of the titanium disc surface; NP, P2, and P10. Contact angles of (**A**) distilled water, (**B**) ethylene glycol. (**C**) Surface energy was calculated using the Owens-Wendt method. The same lowercase letter indicates no significant difference (p > 0.05).

**Figure 3:**
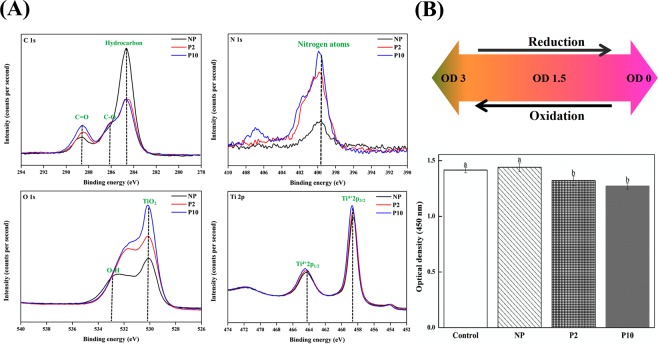
Changes in the chemical composition of the titanium disc surface; NP, P2, and P10. (**A**) XPS analysis of C1s, N1s, O1s, and Ti2p spectra on titanium disc surface following NP (black line), 2 P (red line) and 10 P (blue line). (**B**) Characteristic of oxidation-reduction. The same lowercase letter indicates no significant difference (p > 0.05).

Characteristics of NTAPPJ-treated surfaces were investigated in terms of roughness, surface energy and chemical composition, which may influence biological effects on bacterial cells^19,28^. The topographical characteristics of the NTAPPJ-treated surfaces are related to the physical form of the surface, such as roughness, and the topography of the control and test groups was examined by noncontact 3D optical microscopy. There was no significant difference in the surface roughness parameters, including the Ra and Sa values, between the control and test groups (Fig. 1). The Ra values for NP, P2, and P10 were 0.207 ± 0.004, 0.233 ± 0.009, and 0.229 ± 0.006 µm, respectively (p > 0.05). The Sa values for NP, P2, and P10 were 0.215 ± 0.013, 0.229 ± 0.014, and 0.220 ± 0.011 µm, respectively (p > 0.05). The surface energy is the energy associated with the intermolecular forces at the interface between two media, which were between the NTAPPJ-treated surface and liquid on the surface in this study. The surface energy is typically measured by the contact angle of a liquid on a solid surface, and a low contact angle indicates a high surface energy, while a high contact angle indicates a low surface energy of the solid surface in relation to the liquid. The results of the control and test groups were determined using contact angle measurements with two liquids, distilled water (DW), which is a polar liquid, and ethylene glycol (EG), which is a nonpolar liquid. A significant change in the DW and EG surface contact angles after NTAPPJ treatment was observed in all experimental conditions compared to the untreated control samples (p < 0.05, Fig. 2A,B). The surface energy of the NTAPPJ-treated sample, which was calculated according to Owens-Wendt method, increased more than four-fold compared to that in the untreated samples (Fig. 2C). The difference in the chemical composition of the NTAPPJ-treated and untreated samples was analyzed by X-ray photoelectron spectroscopy (XPS). XPS was used to analyze changes in the chemical composition of the titanium surfaces. First, full-survey XPS scans were analyzed, and C1s, N1s, O1s, and Ti2p were observed. Figure 3A shows the high-resolution XPS spectra for C1s, N1s, O1s, and Ti2p for NP, P2, and P10 surfaces. In terms of the C1s spectrum, the NTAPPJ-treated samples showed a decrease in the amount of hydrocarbon (284.7 eV) molecules and an increase in the amount of carbon atoms related to COOH groups (C_2_ peak) compared to the untreated controls. Hydrocarbons are naturally present on the surface of titanium, even after polishing and ultrasonically cleaning with acetone, ethyl alcohol, and distilled water for 15 min each, and similar results were also observed in other studies^28–30^. The peak near 286 eV in C1s corresponds to the C-O bond, which was a newly formed C1s XPS peak. The C-O bond was observed because the surface of the titanium was cleaned with chemicals, such as ethyl alcohol, and exposure to plasma may lead to bonding between oxygen from ROS with carbon on the titanium surface. The N1s spectrum showed a single peak (N_1_, 399.6 eV) for control samples that corresponded to the free amines (NH_2_), while there was not only an increase in intensity observed in the same peak but also the appearance of two new peaks in the NTAPPJ-treated samples. Furthermore, an N_2_ peak was observed at 400.4 eV that corresponded to nitrogen atoms in amide-N and imide-N functional groups, and an N_3_ peak observed at 401.7 eV corresponded to the protonated amine (NH_3_^+^)^31^. Furthermore, the O1s spectrum for the sample showed a substantial peak with a binding energy at 530.0 eV (O_1_) corresponding to TiO_2_, and the height of this peak was increased in the test groups compared to that in the control group. In particular, NTAPPJ-treated samples showed peaks at 533 eV (O_2_), which was related to hydroxyl (O-H) groups, and the peak height was increased compared with that of the control group^32^. Finally, a Ti2p doublet peak that included both Ti2p 1/2 and Ti2p 3/2 components was observed in the range between 464.3 and 458.7 eV. The gap between the two peaks of the Ti2p doublet was 5.6 eV, and these two peaks corresponded to a normal state of Ti^4+^ in an anatase state in a TiO_2_ layer^29^. The intensity values from the XPS results were not absolute values, and the area under the peak was not analyzed. Nevertheless, the purpose of this analysis was to investigate relative changes in peaks between the control and NTAPPJ-treated samples, while the area under the peak data (results not shown) indicated the same findings as stated above.

To further investigate the chemical changes on the surface of NTAPPJ-treated samples, water soluble tetrazolium (WST) solution was used to investigate oxidative and reductive potential on the surface, where the color of the WST solution changed according to the oxidative or reductive potential on the surface. Figure 3B shows the optical density value of WST solution that was intentionally partially oxidized using magnesium (WST+Mg), and WST+Mg solution was placed on Ti before and after NTAPPJ treatment. The results showed that when the partially oxidized WST solution was placed onto the Ti surface, there was no significant change in the OD value compared to that of the WST+Mg solution, indicating stability on the surface of Ti, which did not allow WST to be reduced or oxidized. However, when WST+Mg solution was placed on the NTAPPJ-treated Ti surface, the OD value was significantly decreased compared with the ODs of the WST+Mg solution (both the WST+Mg solution alone and after placing the solution on Ti). The decrease in the OD value indicated that WST was oxidized. In other words, the plasma-treated Ti surface had a reductive potential, which caused oxidation of the surrounding environment.

### Bacterial adhesion on the NTPPJ-treated surface

**Figure 4:**
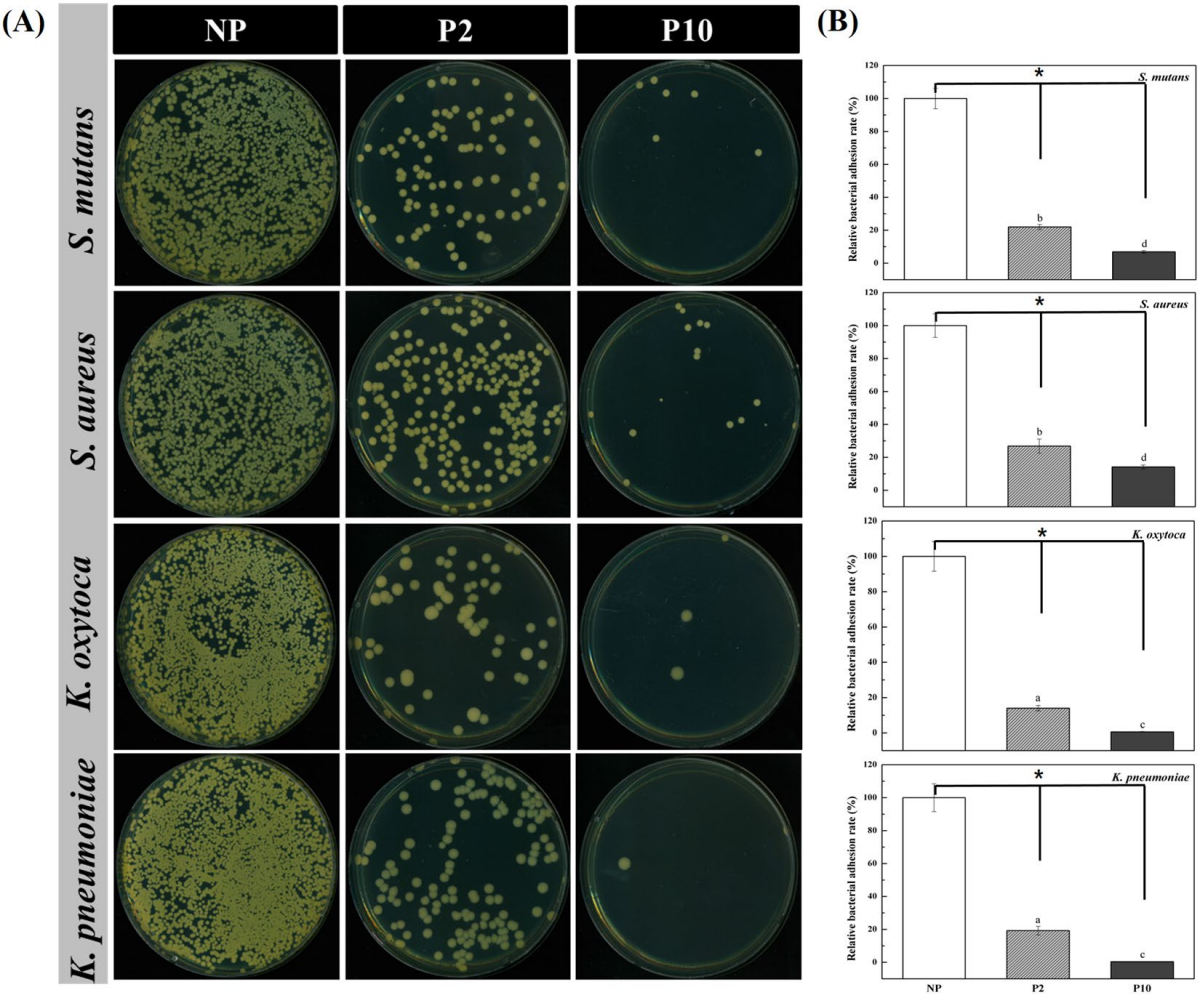
(**A**) Images of colony forming unit (CFU) of four bacterial strains following NP, P2, and P10. (**B**) Relative adhesion rate of *S. mutans*, *S. aureus*, *K. oxytoca* and *K. pneumoniae* on NTAPPJ treated titanium indicated by colony forming unit (CFU). *No differences among the NTAPPJ-treated time groups (p > 0.05). The same lowercase letter indicates no significant difference among the bacteria species groups (p > 0.05).

The adhesion and growth of four strains of bacteria, including *S. mutans, S. aureus, K. oxytoca* and *K. pneumoniae*, on control and NTAPPJ-treated samples were investigated. Both *S. mutans* and *S. aureus* are gram-positive bacteria, while *K. oxytoca* and *K. pneumoniae* are gram-negative bacteria. Colony forming units (CFUs) were counted to analyze bacterial adhesion and growth (Fig. 4). The result showed a significant reduction in bacterial adhesion on the NTAPPJ-treated surfaces, while the lower number of CFUs was observed on the samples with an increase in the duration of NTAPPJ treatment (p < 0.05). When the bacterial adhesion rate of the control was set at 100%, the relative bacterial adhesion rate on NTAPPJ-treated surfaces was significantly decreased for all bacterial strains. For the comparison between strains, the relative bacterial adhesion rates of *S. mutans, S. aureus, K. oxytoca* and *K. pneumoniae* on samples treated with the NTAPPJ for 2 min were 18.99, 26.9, 14.08 and 19.3%, respectively, compared bacterial adhesion rates of the control samples. Additionally, the relative bacterial adhesion rates of *S. mutans, S. aureus, K. oxytoca* and *K. pneumoniae* on samples treated with the NTAPPJ for 10 min were 6.9, 14.2, 0.66 and 0.42%, respectively, compared to the control (Fig. 4B). These results demonstrated that the adhesion rate of the gram-negative bacteria was significantly less than that of the gram-positive bacteria (p < 0.05).

### Biofilm formation ability on NTAPPJ-treated surfaces

**Figure 5:**
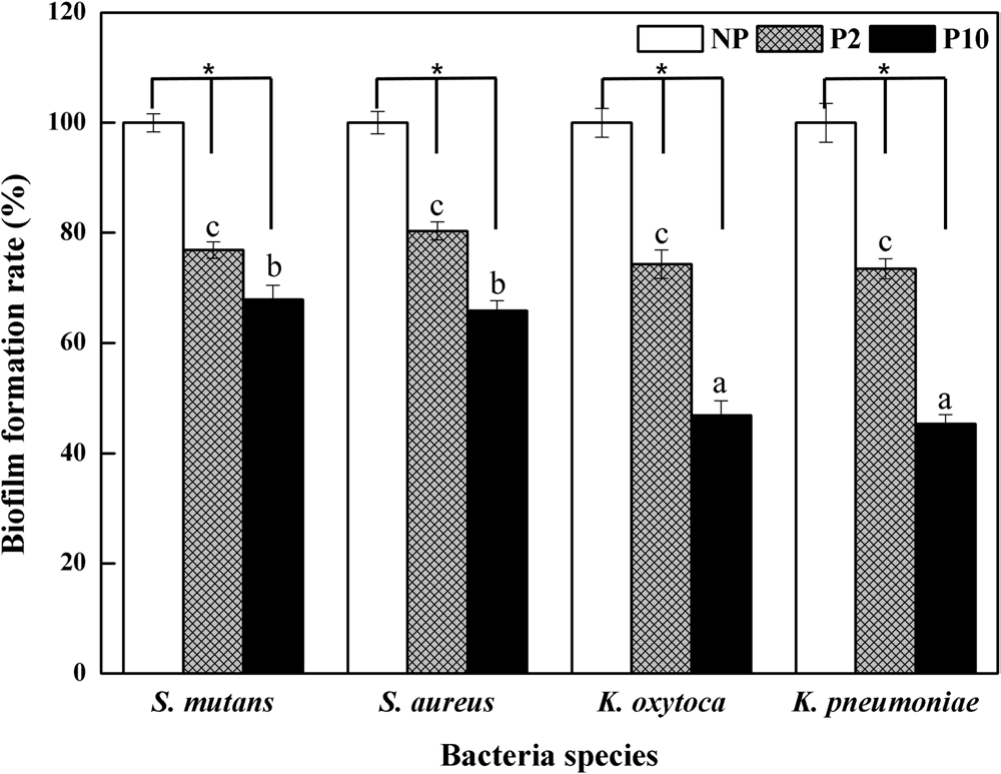
Changes in rate of biofilm formation ability by four bacteria species. *No differences among the NTAPPJ-treated time groups (p > 0.05). The same lowercase letter indicates no significant difference among the bacteria species groups (p > 0.05).

Crystal violet dye was used to analyze the ability to inhibit biofilm formation, because dye binding is influenced by diffusion as well as morphological and physiological differences in individual cells^33^. The results of the crystal violet assay for the four different bacteria are shown in Fig. 5. In the control groups, the degree of biofilm formation was significantly greater (p < 0.05) than in the test groups for all four bacteria. In addition, a longer NTAPPJ treatment duration (P10) generally resulted in a lower biofilm formation rate than samples exposed to NTAPPJ for shorter durations (P2).

In terms of the biofilm formation rate between different bacteria, the rates following 2 min of NTAPPJ exposure on Ti for *S. mutans, S. aureus, K. oxytoca* and *K. pneumonia* were 76.863 ± 1.511, 80.325 ± 1.649, 74.277 ± 2.584 and 73.442 ± 1.812%, respectively, compared to the control. The results indicated that there were no significant differences in the biofilm formation rate between the bacterial strains (p > 0.05). However, following 10 min of NTAPPJ exposure on Ti, the rates for *S. mutans, S. aureus, K. oxytoca* and *K. pneumonia* were 67.866 ± 2.605, 65.853 ± 1.781, 46.887 ± 2.673 and 45.411 ± 1.658%, respectively, compared to the control. The results showed that there was a significantly lower biofilm formation rate for gram-negative bacteria than for gram-positive bacteria following 10 min of NTAPPJ exposure on Ti (p < 0.05).

### Bacterial viability on NTAPPJ-treated surfaces

**Figure 6:**
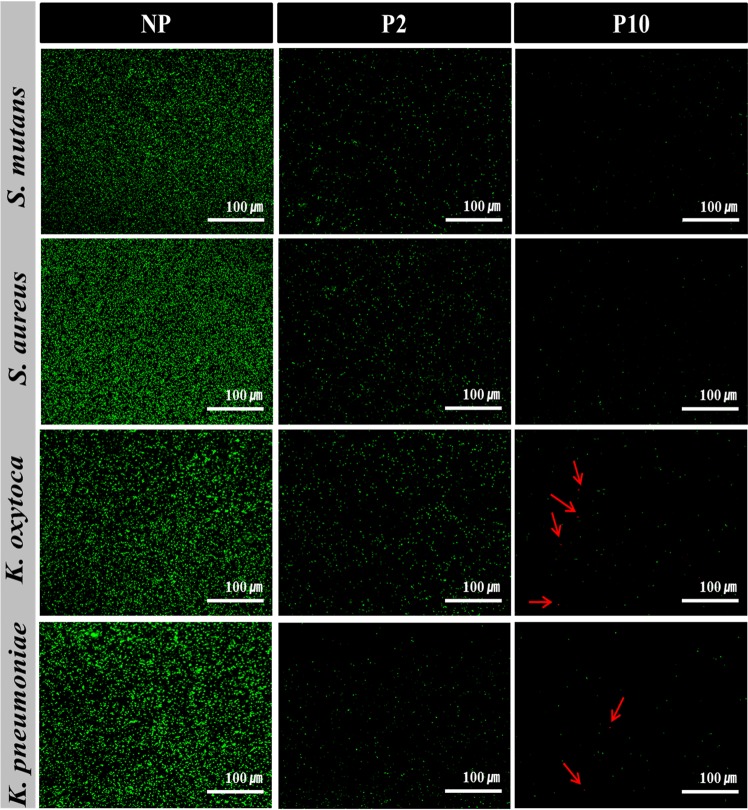
Changes in bacterial viability on the titanium disc surface; NP, P2, and P10. Fluorescent images showing the live (green) and dead (red, arrow) stained bacteria adherent to titanium disc (scale bar = 100 µm).

The viability of four species of bacteria was determined by a BacLight LIVE/DEAD assay kit. Stained control and test group bacteria were examined under a confocal laser scanning microscope (Fig. 6). This assay used two DNA intercalating dyes, SYTO9 and propidium iodide (PI)^34^. Green fluorescent SYTO9 stains viable cells by penetrating all the membranes, whereas red fluorescent PI only penetrates permeabilized membranes and stains cells with a damaged cytoplasmic membrane^35^. The number of viable bacteria, stained with a green fluorescent stain, was greater in the control group than in the test group. After NTAPPJ treatment, the amounts of viable bacteria were clearly lower in the test groups, which also confirmed the above results of bacterial adhesion, growth and biofilm formation. Interestingly, among attached gram-negative bacteria, a few dead bacteria were observed on surfaces treated with NTAPPJ for 10 min (red arrow on Fig. 6).

### Bacterial morphology on NTAPPJ-treated surfaces

**Figure 7:**
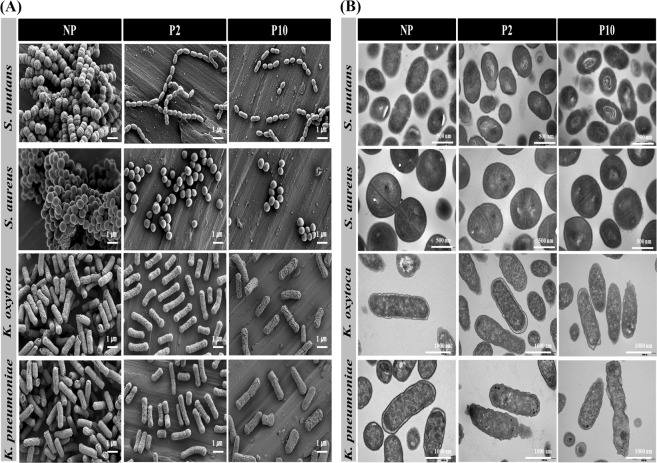
Changes in bacterial morphology on the titanium disc surface; NP, P2, and P10. (**A**) Field-emission scanning electron microscopy images of bacteria adherent to titanium disc following NP, P2, and P10 (scale bar = 1 µm). (**B**) Transmission electron microscopy images of bacteria adherent to titanium disc following NP, P2, and P10 (scale bar = 500 nm, scale bar = 1000 nm).

Scanning electron microscopy (SEM) analysis of bacteria was carried out to observe any morphological changes (Fig. 7A). Generally, connected and aggregated bacterial cells were observed on the control surfaces, while there were more disconnected and malformed bacterial cells on the NTAPPJ-treated surfaces. Notably, the structure of the streptococcal chains of *S. mutans* changed from a long-chain configuration to a short-chain configuration.

To further investigate the changes in the bacteria before and after NTAPPJ treatment, transmission electron microscopy (TEM) images were examined (Fig. 7B). The cell surfaces of gram-positive bacteria (*S. mutans* and *S. aureus*) on untreated surfaces were observed to have normal cellular morphology with homogeneous electron density in the cytoplasm. Their cell walls and membranes remained intact, showing a well-preserved peptidoglycan layer and cytoplasmic membrane^36^. In contrast, bacterial cells on NTAPPJ-treated surfaces showed noticeable damage with a disorganized cell surface morphology and heterogeneity in electron density on the cytoplasm^37^. Cellular damage was clearly observed with cytoplasmic release. Similar results were obtained from the TEM images of the gram-negative bacteria, *K. oxytoca* and *K. pneumoniae*. The untreated bacterial cells showed an intact cell membrane and wall with a uniform electron density, while the internal cellular structures were observed to be normal, showing a multilayered cell surface consisting of an outer membrane, a peptidoglycan layer in the periplasmic space, and a cytoplasmic membrane^38^. However, bacteria on the NTAPPJ-treated surface were severely damaged following the disorganization of the bacterial cytomembrane and leakage of intracellular content. Cellular degradation was also accompanied by electron-translucent cytoplasm and cellular disruption in the damaged cells.

In addition to the similar morphological changes on all types of bacteria, slight differences were also observed between gram-positive and gram-negative bacteria on NTAPPJ-treated surfaces. *S. mutans* and *S. aureus* retained their integrity, the amounts of the electron-dense granules inside the cells were smaller, and the electron-light region was comparatively darker than that of *K. oxytoca* and *K. pneumoniae*^36^. All of these results suggested that gram-positive bacteria may have a stronger defense system against plasma treatment, which seems to be related to cell wall structure^37,38^.

## Discussion

In modern dentistry, titanium implants have an essential role in the re-establishment of aesthetics and chewing function following tooth loss. However, failure of implants due to infection remains the most common issue and is a continuous challenge^39^. The success of dental implantation depends on antibacterial activity and the characteristics of the implant surface^6^.

Bacterial adhesion to titanium implants has previously been shown to be related to critical factors, such as surface roughness, surface free energy, and chemical composition^28,40^. Therefore, understanding how NTAPPJ treatment affects these surface properties is important for predicting clinical outcomes. NTAPPJ treatment was shown to affect the surface chemistry and energy, although it did not affect the surface roughness of titanium surfaces. An increase in the surface roughness leads to enhanced bacterial adhesion, especially within surface irregularities^40^. The results of this study showed that there was no significant difference in the surface morphology and the 2D and 3D surface roughness values (Ra and Sa) between the NTAPPJ-treated and untreated samples (Fig. 1).

However, both contact angle and XPS analysis showed significant changes in the surface energy and chemical compositions of NTAPPJ-treated samples (Figs 2 and 3A). NTAPPJ treatment led to an increase in the levels of hydroxyl-related ions, such as OH^−^ and COOH^−^ (Fig. 3A). Additionally, NTAPPJ treatment led to a decrease in hydrocarbon content. These chemicals improve the hydrophilicity of a surface^30^. Changes in surface chemistry can change the hydrophilicity as well as the surface energy, which are related to the effectiveness of bacterial adhesion^41^. These results indicated that the NTAPPJ treatment was effective in controlling the surface chemistry, including the surface energy, without affecting the physical properties of the materials. In addition to these effects on titanium surfaces, the influence of oxidation-reduction changes was investigated. Bacterial adhesion is affected by oxidation-reduction changes in the environment^42^. A WST assay was used, which was originally intended to be used as a sensitive colorimetric assay for the determination of cell viability in cell proliferation and cytotoxicity assays^43^. WST salt is reduced by dehydrogenase activities in cells, resulting in a yellow-color formazan dye, which is soluble in culture media. WST produces a water-soluble formazan dye upon reduction in the presence of an electron carrier^44^. Additionally, WST reactions are reversible and can produce oxidation and reduction agents.

Magnesium is a strong reducing agent. Its oxidation could cause the reduction of surrounding materials. In the process of magnesium oxidation, Mg^2+^ ions and two electrons are released, as shown in the equation below.

1 $${\rm{Mg}}\leftrightarrow {{\rm{Mg}}}^{2+}+2{{\rm{e}}}^{-}$$

WST could be reduced by a few electrons to WST with a change in color (reduced WST). WST reactions are reversible, and reduced WST could be oxidized to normal WST. Since normal WST cannot continue to be oxidized (no color change), the OD value of the reduced WST can be up to 3. Therefore, to determine whether the plasma is capable of oxidation or reduction, we used a magnesium block to maintain the OD value of WST at 1.5.

2 $${{\rm{WST}}}_{{\rm{0}}}+{{\rm{ne}}}^{-}\leftrightarrow {{\rm{WST}}}_{1.5}+{{\rm{ne}}}^{-}\leftrightarrow {{\rm{WST}}}_{{\rm{3}}}$$

If the NTAPPJ-treated surface (NTAPPJS) has oxidizing potential, it can cause WST reduction, and the OD value will be increased.

3 $${{\rm{WST}}}_{1.5}+{\rm{NTAPPJS}}\to {{\rm{WST}}}_{{\rm{3}}}$$

However, if the NTAPPJS has a reductive potential, it can cause WST oxidation, and the OD value will be decreased.

4 $${{\rm{WST}}}_{1.5}+{\rm{NTAPPJS}}\to {{\rm{WST}}}_{0}$$

The results of this study indicated that the NTAPPJ-treated Ti surface had reductive potential, and therefore, the surface can oxidize the surrounding matter.

**Figure 8:**
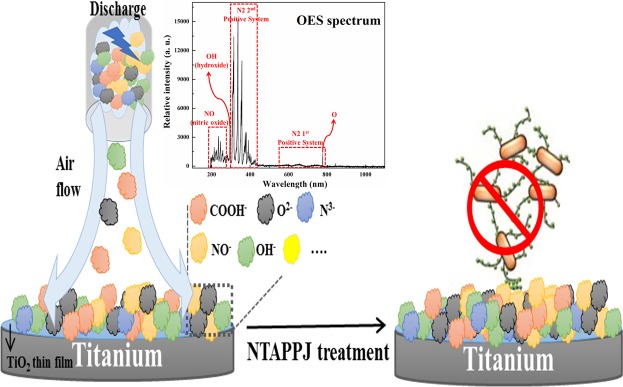
Schematic illustration of a summary of possible events in bacteria present on the NTAPPJ-treated titanium surface.

Titanium is a reactive metal that is rendered corrosion resistant. When titanium was exposed to air, a thin oxide layer was formed on the titanium surface because of the high affinity for the oxygen content in titanium^29^. Therefore, titanium biomaterials have chemical stability and biocompatibility for *in vivo* applications by acting as a passive layer^1^. After treatment, TiO_2_ is a wide band gap semiconductor that could be modified by ions, such as COOH^−^, NO^−^, OH^−^, N^3−^ and O^2−^, which would consequently result in surfaces as shown in the XPS analysis (Fig. 3A). Most of the species and elements indicated in the XPS results are attributed to these species from NTAPPJ air flowing plasma and their RONS species. These species and elements were easily observed and demonstrated by the optical emission spectrum of the air plasma jet (Fig. 8)^45,46^. There is evidence indicating that these free radicals may be successfully used in oxidation-reduction chemistry, affecting the adhered bacteria^47^. Since the plasma acted as a reducing agent (Fig. 3B), it oxidized the surrounding environment. As a result, it was assumed that bacteria were adversely affected (Fig. 8)^48^.

UV production is accompanied by plasma generation, and UV produces reactive chemical species on the titanium surface, killing bacteria by acquired redox chemistry^25^. However, a previous study demonstrated that this type of an effect by UV irradiation has a limited effect on bacterial adhesion because the effect remains on the material surface for a shorter period of time^32^. Additionally, a relatively fast effect can also be obtained using an NTAPPJ as a result of radicals diffusing deeply inside the pores, leading to lethal oxidative damage^49^. Therefore, the NTAPPJ has been indicated as an effective functionalizing method for titanium surfaces compared to UV irradiation^28^.

The human oral cavity is colonized by more than 1000 bacterial species^50^. Among these species, we used four kinds of bacteria, including gram-positive and gram-negative bacteria, which were oral bacteria associated with dental diseases and periodontal pathogens from clinically relevant strains obtained from the Korean Collection for Oral Microbiology^51^. These four kinds of bacteria have been suggested as the most commonly observed species in the oral cavity, where there are difficulties in suggesting which kind of bacteria is more abundant. Nevertheless, all species have been speculated to be responsible for oral disease, and understanding the effects of plasma on these bacteria may provide information that is applicable to other oral bacteria^50,51^. To the best of our knowledge, this study is the first to further investigate the underlying mechanisms of NTAPPJ treatment against gram-positive and gram-negative bacteria by a series of analyses. The difference in the adhesion of the four strains of bacteria on the surface is shown in Fig. 4. From the limited number of tested bacteria, the results confirmed a significant reduction in bacterial adhesion on the NTAPPJ-pretreated surfaces (p < 0.05). In addition, the NTAPPJ treatment inhibited the growth of gram-negative bacteria, which included *K. oxytoca* and *K. pneumonia*, much more than gram-positive bacteria, which included *S. mutans* and *S. aureus*. Similar results were observed with the biofilm formation rate, where lower rates were observed with gram-negative bacteria, which included *K. oxytoca* and *K. pneumonia*, than the gram-positive bacteria, which included *S. mutans* and *S. aureus*, when cultured on NTAPPJ-treated Ti for 10 min (Fig. 5). Previous studies have clearly shown that gram-negative bacteria are more susceptible to plasma than gram-positive bacteria^52,53^. Fluorescent imaging of viable bacteria also confirmed these results (Fig. 6). It has been previously shown that gram-negative bacteria are mechanically weaker than gram-positive bacteria^37^. This was possibly due to the thickness of the peptidoglycan layer in the bacterial cell wall, which may reduce the antibacterial effects; this result was consistent with the results of other studies^35,37,54^. This study demonstrated that these results were reproducible with NTAPPJ-pretreated titanium. Indeed, TEM showed that gram-negative bacteria had disrupted membranes compared to gram-positive bacteria when they were cultured on NTAPPJ-treated Ti surfaces (Fig. 7B). Nevertheless, the exact mechanism related to interactions between NTAPPJ-treated surfaces and bacterial cell walls needs to be studied further.

In this study, an NTAPPJ supplied with compressed air was used because it may be desirable to use gases that are inexpensive and easily available in the clinic. Additionally, a duration of 10 min was shown to be sufficient to have an antibacterial effect, which confirmed previous findings^32^, while the effect was observed to last up to 24 h. Surfaces of implants are susceptible to bacterial colonization during the initial 6 h after implantation^39^. Therefore, the antibacterial effects on the first day are critical for successful implantation. The results of this study confirmed that with such a short exposure duration of 10 min, effective antibacterial properties can be achieved.

Within the limitations of this study, we have demonstrated the following results.

1. The changes in titanium surface properties, such as surface energy, chemical composition and reductive potential induced by NTAPPJ treatment, lead to a significantly lower number of adhered bacteria and a lower biofilm formation rate.
2. The effect was observed to be greater with gram-negative bacteria, which was likely due to the difference in the bacterial wall structures between gram-positive and gram-negative bacteria influenced by the oxidation of the NTAPPJ-treated surface.

This study considered the antibacterial effects of NTAPPJ treatment on the titanium surface, which was carried out using *in vitro* studies. The limitation of this study is that the test results may differ in clinically relevant oral environments. Seo *et al*. examined the antibacterial activity of non-thermal atmospheric pressure plasma in artificial saliva, attempting to mimic some of the *in vivo* conditions. The obtained results indicated that plasma treatment may be more effective in a physiological oral environment with saliva than under certain experimental conditions^43^. In this study, the bacterial test method and the plasma treatment method were similar, although there was a difference in using broth without artificial saliva. Nevertheless, the experimental results showed that the antimicrobial effect of the plasma treatment was similar. These findings could encourage the use of NTAPPJ functionalization of dental implants in clinical settings.

Additionally, an *in vitro* study is a prerequisite to ensure the long-term survival of titanium implants, where *in vivo* experiments or clinical studies may be required to confirm whether the *in vitro* results are applicable to actual clinical situations.

Despite the limitations of the present study, plasma jet applications are used in multidisciplinary research in dentistry to address problems. We demonstrated that plasma treatment decreased not only bacterial adhesion but also bacterial viability when used indirectly via the treatment of a titanium surface. Therefore, these results could be useful not only for preventing infection before implantation but also for reducing bacterial implant contamination during the surgical process. Our study offers an in-depth understanding of NTAPPJ treatment for the inhibition of bacterial adhesion on titanium surfaces. Thus, NTAPPJ treatment could significantly reduce dental implant-related diseases and implantation failure.
